# The Importance of Pedestrian Network Connectivity for Adolescent Health: A Cross-sectional Examination of Associations between Neighbourhood Built Environments and Metabolic Health in the Pacific Islands Families Birth Cohort Study

**DOI:** 10.3390/ijerph16183375

**Published:** 2019-09-12

**Authors:** Melody Smith, Vlad Obolonkin, Lindsay Plank, Leon Iusitini, Euan Forsyth, Tom Stewart, Janis Paterson, El-Shadan Tautolo, Fa’asisila Savila, Elaine Rush

**Affiliations:** 1 School of Nursing, the University of Auckland, Auckland 1142, New Zealand; 2 School of Sport and Recreation, Auckland University of Technology, Auckland 1142, New Zealand; 3 Department of Surgery, the University of Auckland, Auckland 1142, New Zealand; 4 School of Public Health and Psychosocial Studies, Auckland University of Technology, Auckland 1142, New Zealand; 5 School of Environment, the University of Auckland, Auckland 1142, New Zealand; 6 School of Population Health, the University of Auckland, Auckland 1142, New Zealand

**Keywords:** moderate-to-vigorous physical activity, diabetes, body composition, fat free mass

## Abstract

The research aim was to investigate associations between objectively-assessed built environment attributes and metabolic risk in adolescents of Pacific Islands ethnicity, and to consider the possible mediating effect of physical activity and sedentary time. Youth (*n* = 204) undertook a suite of physical assessments including body composition, blood sampling, and blood pressure measurements, and seven day accelerometry. Objective measures of the neighbourhood built environment were generated around individual addresses. Logistic regression and linear modelling were used to assess associations between environment measures and metabolic health, accounting for physical activity behaviours. Higher pedestrian connectivity was associated with an increase in the chance of having any International Diabetes Federation metabolic risk factors for males only. Pedestrian connectivity was related to fat free mass in males in unadjusted analyses only. This study provides evidence for the importance of pedestrian network connectivity for health in adolescent males. Future research is required to expand the limited evidence in neighbourhood environments and adolescent metabolic health.

## 1. Background

### 1.1. Prevalence and Burden of Metabolic Disease Risk Factors

Adolescent metabolic syndrome is a global and growing health problem [1]. Complications are substantial, and development can be especially rapid when onset of metabolic diseases such as type 2 diabetes mellitus is early in life [2]. While definitions differ, metabolic syndrome is typically characterised by suboptimal levels of triglycerides, high-density lipoprotein cholesterol (HDL-C), blood pressure, glucose, and body composition. Obesity is the prevailing risk factor for metabolic syndrome in adolescents [3,4].

Disparities exist across a number of metabolic indicators for Pacific peoples living in New Zealand, including higher rates of child and youth obesity (29.7% compared with 10.8% in the total New Zealand population), and higher estimated rates of diabetes prevalence (10.5% compared with 6.0% in the total population) [5]. Moreover, the estimated prevalence of diabetes continues to increase at a faster rate in Pacific peoples than the total New Zealand population [5]. Understanding factors to inform early intervention in this population is imperative to reduce substantial future burden of disease.

### 1.2. Physical Activity and Metabolic Risk

Physical activity, particularly of a moderate-to-vigorous nature (MVPA), has a crucial role in reducing metabolic risk. MVPA has a clear effect on maintaining healthy body composition [6,7,8]. Links between MVPA and broader metabolic health measures have also been established. For example, using a composite measure of “normal” versus “at risk” or “high risk” levels of triglycerides, HDL-C, systolic and diastolic blood pressure, glucose, and homeostatic model of insulin resistance (HOMA-IR), associations with accelerometer-derived MVPA were observed in a large international study of children and youth aged 3–18 years from Denmark, Estonia, Portugal, and the United States [9]. Differential relationships have been observed between different physical activity measures (i.e., self-report surveys and accelerometry) and biomarkers (i.e., systolic and diastolic blood pressure, lipids, percent body fat, and body mass index (BMI) percentile) [8], demonstrating the need for robust, objective measures where possible.

Metabolic risk and physical activity track from adolescence to adulthood, with demonstrated longitudinal associations between activity and metabolic outcomes over these life stages [10,11,12]. Adolescence is an essential life stage for cementing health-promoting behaviours for short-term and long-term health gains. Yet, this period is a time of significant decreases in, and low levels of, physical activity [13,14,15]. In New Zealand, approximately 38–39% of adolescents are sufficiently active for health [16]. Evidence suggests Pacific youth living in New Zealand are less physically active than their non-Pacific counterparts [17].

### 1.3. Ecological Models, the Built Environment, and Physical Activity

Socio-ecological models posit that behaviours are impacted at varying levels, including the individual (e.g., demographic characteristics), family, and built environment [18]. The built environment has received much attention in this context, with a clear impact on physical activity in people of all ages [19,20]. Less is known with regard specifically to adolescents. In the Healthy Lifestyle in Europe by Nutrition in Adolescence (HELENA) study including 3528 adolescents aged 12.5–17.5 years [21], heavy neighbourhood traffic (negative) and a secure bicycling or walking route from home to school (positive) were significantly associated with physical activity. One study that has used global positioning systems (GPS) units and accelerometers in 293 US adolescent females aged 15–18 years revealed the odds of higher intensity physical activity were higher in places with parks, schools, and high population density; and lower in places with more roads and food outlets [22]. Conversely, another study of adolescent girls in the United States showed accessibility measured around the school (number of food stores near school) was positively associated with physical activity [23].

In the Built Environment and Physical Activity in New Zealand Adolescents (BEANZ) study (1.5% of whom identified as being of Pacific Islands ethnicity), a combined index of residential density and number of parks within 2 km from home was associated with MVPA [24]. Specifically comparing those in the minimum and maximum observed values of the index, approximately 28 min more MVPA per day was observed. In the Kids and the City study of New Zealand children aged 9–13 years (36% of Pacific Islands ethnicity), the ratio of high speed to low speed roads and improved streetscape for active travel were related to weekday MVPA only [25].

Connectivity implies greater ease of mobility in and around neighbourhoods. For the most part, street network connectivity has been the focus in environment and activity research, due to its comparative ease of calculation. Using street connectivity only can introduce bias and understate the connectivity of an environment at the human scale (rather than that of a motorised vehicle). This measure can be susceptible to errors in road classification, and can result in the inclusion of networks that are unsuitable for human movement, such as motorways [26]. Despite these limitations, consistent links between street connectivity and physical activity in adults have been observed [27,28]. Less is known regarding younger populations; previous research with adolescents has shown positive links between activity and walkability indices (including street connectivity) [29]. With younger children (aged 9–13 years) in New Zealand, street connectivity was associated with increased active travel (e.g., walking or cycling for transport) on weekdays and weekend days [25]. 

In the context of understanding active behaviours in the neighbourhood context, pedestrian networks improve on street networks by including factors such as informal paths, laneways, and pedestrian bridges [30,31]. This increased resolution improves sensitivity and specificity by characterising the built environment in a manner that would be expected to relate to physical activity in the neighbourhood setting. In a representative random sample of 1209 Northern Ireland adults aged 16 years or over, Cruise et al. [26] found street and pedestrian network connectivity measures were comparably associated with self-reported active travel, with only marginal differences in adjusted model fit. To some extent these marginal differences may be due to the typology of the environment. Chin et al. [30] discovered that in four Perth neighbourhoods, traditional neighbourhoods had considerably higher street network connectivity than conventional neighbourhoods. However, when pedestrian network connectivity was taken into account, the differences between the neighbourhoods were often negligible (but still in favour of traditional neighbourhoods). 

### 1.4. Built Environment and Metabolic Risk Factors

Associations between the neighbourhood built environment and metabolic risk factors in adults are clear. In their review, Malambo et al. [32] reported high walkable environments (e.g., environments characterised by greater street network connectivity, residential density, and ease of access to amenities) were associated with lower blood pressure, BMI, diabetes mellitus, and metabolic syndrome. A cross-sectional study of 78,023 Canadian adults revealed significant relationships between walkability (assessed using the Walk Score index) and lower BMI, systolic blood pressure, diastolic blood pressure, and HbA1c (glycated hemoglobin), and higher HDL-C [33]. Longitudinal results from the CARDIA Study of 1079 adults showed a one standard deviation increase in walkability (index comprising population density, street connectivity, food, and physical activity resources) was related to a 0.81mmHg decrease in systolic blood pressure and a 7.36% increase in C-reactive protein [34]. 

Our earlier work with 2020 New Zealand adolescents and adults aged 15–65 years showed associations between street connectivity, destination accessibility, streetscape, and dwelling density and body size [35]. After adjusting for individual characteristics, neighbourhood preference, and neighbourhood-level deprivation; street connectivity and destination accessibility were associated with reduced BMI and waist circumference (ranging from −1.27% to −2.29% for a one SD change in each built environment variable). Higher quality streetscape was also associated with reduced BMI, but this relationship did not hold true for waist circumference. Dwelling density was associated with reduced waist circumference (−1.97%, *p* = 0.004), and this relationship neared significance for BMI (−1.10%, *p* = 0.061). Importantly, objectively-assessed physical activity (mean accelerometer counts per hour) had a significant mediating effect on the relationship between body size and street connectivity and destination accessibility (explaining between 10–15% of the total effect). No mediating effect of objectively-assessed sedentary time was observed.

Conversely, one study found most built environment features were not associated with BMI z-score in 1034 US adolescents but that differences by ethnicity existed [36]. There is some evidence for geographic variation in levels of HDL-C in children aged 6–15 years [37]. Overall, the evidence base related to adolescent metabolic risk and built environments is sparse (especially with regard to risk factors other than body size) and inconsistent. 

### 1.5. Advances in Objective Measures of the Built Environment

An important challenge in the field of environments and health is the use of environmental measures that are objective, and sensitive and specific to the population of interest [38,39]. A number of advancements in environment and health research specific to child and youth populations have been made to overcome these challenges. These include the development of measures of pedestrian network connectivity and potential exposure to traffic (using road hierarchy as a proxy) [40], and distance decay approaches to measuring access to destinations [41]. Neighbourhood topography could encourage or discourage participation in physical activity, and could also play an important role in metabolic health (through higher intensity activity accumulated when encountering greater slopes). This variable is yet to be examined in relation to adolescents’ physical activity or metabolic health. 

### 1.6. Research Gaps and Study Aim

There is a growing evidence base for the link between the built environment and body size in adolescents but less is known for other metabolic risk factors. To the authors’ knowledge, no research has considered the possible mediating effect of objectively-assessed physical activity or sedentary time on this relationship. There is a dearth of evidence related to Pacific peoples, a population exhibiting extremely high rates of metabolic risk factors compared to their non-Pacific peers. There is a need to improve knowledge in the field of environments and health through use of sensitive and specific measures of the neighbourhood built environment. The aim of this study was to investigate associations between objectively-assessed built environment attributes and metabolic risk factors in adolescents of Pacific Islands ethnicity residing in Auckland, New Zealand, and to consider the possible mediating effect of moderate-to-vigorous physical activity and sedentary time in these relationships. 

## 2. Methods

### 2.1. Protocol

The methods for the Pacific Islands longitudinal birth cohort study and the current sub-study have been described previously [42,43,44]. Briefly, the full cohort were invited to participate in the 14-year wave of the study in 2014–2015 (i.e., when the participants were aged 14–15 years). Approximately half-way through the 14-year wave, a subset of the cohort participants was invited to participate in a nested study involving a suite of objective physical assessments. Stratified sampling of the full cohort determined the nested study participants to ensure their characteristics were relatively evenly spread for sex and body weight decile (using body weight data from the 11 year wave). Sub-study participants were transported to and from the University of Auckland Body Composition Laboratory, based at Auckland City Hospital, for height and weight measurements, body composition by dual-energy X-ray absorptiometry (DXA), blood sampling, and blood pressure measurements. Participants were instructed to arrive at the laboratory in the morning in a fasted state (no food overnight or in the morning) and were provided breakfast and a gift voucher to acknowledge their time and contribution at the completion of the assessment. Participants’ home and school addresses were also confirmed at this time. Accelerometers on elasticated belts were provided at this time with instructions on how to wear the belt over the next seven consecutive days. Accelerometers were collected by a research assistant approximately eight days after the laboratory visit. Measures specific to the current study are detailed below.

Ethical approval for the full cohort study was obtained from the Southern Health and Disability Ethics Committee on 4 December 2013 (ref. 13/STH/159) and for the nested sub-study from the Central Health and Disability Ethics Committee on 28 July 2014 (ref. 14/CEN/108).

### 2.2. Measures

#### 2.2.1. Individual and Household Demographic Factors

Biological sex was recorded at birth. Household socio-economic status (SES) was assessed using the New Zealand Index of Socioeconomic Deprivation for individuals (NZiDep), asked of primary caregivers in their 14-year wave interview [45]. 

#### 2.2.2. Metabolic Risk

Metabolic risk factors for inclusion in this study were drawn from the International Diabetes Federation criteria for metabolic syndrome for youth aged 10 to 15 years of age [46]. Recognising the prevailing role of body composition in metabolic risk, fat free mass was also examined.

##### Physical Assessments

*Waist circumference* was measured at the narrowest point between the ribs and hip bones. The participant stood tall and measurements were on exhalation. Measures were in duplicate to the nearest 0.1 cm and were repeated if they differed by more than 0.5 cm. The average was determined.

*Blood pressure* was measured after at least five minutes of sitting using an automated oscillometric device (Omron HEM-7200, Omron Healthcare, Kyoto, Japan). Measures were in duplicate and averaged. Measures were repeated if systolic or diastolic differed by more than 10 mmHg. 

*Fat-free mass* (FFM) was derived from DXA (model iDXA, software v.15, GE-Lunar, Madison, Wisconsin, USA) measures of total body fat, lean soft tissue, and bone mineral content as the sum of lean soft tissue mass and bone mineral content and treated as a continuous measure.

##### Blood Biomarkers

During the laboratory visit participants provided a 15 mL fasting venous blood sample. Samples for glucose and lipid profile (as well as other measures not specific to this study) were sent to an accredited laboratory (LabPLUS) for processing on the day of data collection. Standard automated procedures with photometric and electrochemiluminescence detection methods were employed (Cobas C8000 modular analyser, Roche Diagnostics).

Metabolic syndrome criteria were identified based on the International Diabetes Federation (IDF) criteria for ages 10 to 15 years [46] as follows: Triglycerides ≥ 1.7 mmol/L, HDL-C ≤ 1.03 mmol/L, systolic blood pressure ≥ 130 or diastolic blood pressure ≥ 85 mmHg, and fasting blood glucose ≥ 5.6 mmol/L. High waist circumference was determined using sex-specific 90th percentile thresholds for waist circumference established in the Third National Health and Nutrition Examination Survey for all adolescents measured at 14 years of age [47]. 

#### 2.2.3. Built Environment

Objective built environment measures were calculated in ArcGIS 10.2 (ESRI, Redlands, CA, USA), as detailed below. Variables were calculated around individual residential addresses using street and pedestrian network 1200 m buffers. One of two different street networks were used, depending on the variable being calculated. When measuring traffic speed exposure, a road network identifying the speed limit for each road was used. For all other variables, a pedestrian network of all non-motorway roads, and supplemental pedestrian pathways was used. Motorways were removed as they are not a part of the pedestrian network.

##### Walkability Measures

**Figure 1:**
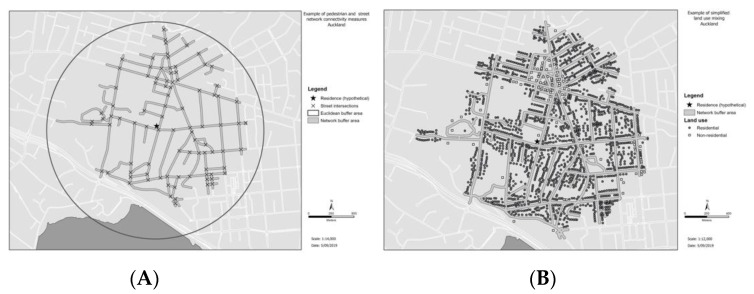
Graphical examples of walkability measures used in the current study for a hypothetical residential address. (**A**) depicts pedestrian network and street connectivity, (**B**) depicts a simplified (binary) example of land use mix, using residential and non-residential land use. Note that non-residential land use mix has not been separated into the individual categories used in this study for ease of interpretation.

Figure 1 provides examples of walkability measures employed in the current study.

*Net residential density* was calculated as the ratio of housing units to the area of land devoted to residential land use. Density values were calculated by dividing the number of joined points by the area of the network buffer. 

*Land use mix* was calculated using the Shannon entropy index. The ‘Calculate Species Diversity Index for Polygons’ tool within the Marine Geospatial Environment Toolbox [48] was used to implement this measure. This tool assesses the degree of land use mixing around each participant’s residence and assigns an entropy score, with a higher score indicating a greater degree of land use mixing.

*Street network connectivity* was measured by calculating the density of intersections around each participant’s residence. For the purpose of this variable, ‘intersections’ were defined as a point at which three or more walkable segments intersect. The number of intersections around each residence were first counted. The number was then divided by the area of the network buffer to provide a density value.

*Pedestrian network connectivity* was measured as the ratio of reachable pedestrian network area to the maximum possible (Euclidean) area within a given distance of each residence [40] and expressed as a percentage. The network buffer area (NBA) was generated for each input point then the Euclidean buffer area (EBA) was generated around each point. The network connectivity variable was calculated as NBA/EBA, with a higher ratio indicating that a greater proportion of the maximum possible distance can be reached through the pedestrian network, and, therefore, a more connected pedestrian network.

##### Accessibility Measures

*Accessibility to open spaces* was assessed by measuring the ratio of area of open space to the neighbourhood area (using the 1200 m boundary) [49] expressed as a percentage. The simulated park entry points from the land use data were extracted into a separate file, following which a tabular join was used to link the park area values from the original polygon features with the points representing the park. The spatial join model was used to count the number of intersecting park points, and to summarise the open space area (OSA) value. The network buffer area value had already been calculated, with the value again used here. The ratio of open space to the defined distance boundary was thus calculated as OSA/NBA. A higher ratio indicates that more open space can be reached, with ratios >1 indicating that the area of open space exceeded the defined boundary. 

*Accessibility to destinations* in the neighbourhood (e.g., shops and doctors) was assessed using a spatially derived, objective destination accessibility index. A description of its calculation is provided elsewhere [41]. Briefly, the index was created for the BEANZ study [50] using data on global positioning systems-derived adolescent travel frequency to destinations. Different types of destinations were weighted according to their visit frequency, with more frequently visited destinations weighted higher. An exponential distance decay function adjusts the weighting of each destination based on its distance from the participant’s residence. Destinations further from the residence are weighted lower. Scores range from 0 to 100, with a maximum score obtained if all types of destination fall within 400 m of the residence.

##### Traffic Safety

*Traffic speed exposure* was calculated as the ratio of the cumulative length of high speed (≥60 km/hour) to low speed roads (<60 km/hour) around each residence [25,40] and expressed as a percentage. For each residential address, the defined network buffer was first created. This buffer was used as the basis to first clip the low speed roads, and then summarise the total length of the clipped roads, thus providing the total low speed length (LSL). This process was then repeated for the high speed roads to calculate the high speed length (HSL). A tabular join was used to merge the individual low and high speed files with the base input points. In this merged table, the traffic speed exposure ratio was calculated as HSL/LSL. Higher ratios indicate a less walkable environment, in other words there were more high speed roads within the defined distance than low speed roads. 

##### School Route Variables

*Distance to School* was done by measuring the shortest pedestrian route from each participant’s residence to their stated school was achieved through the use of a paired origin/destination matrix. In the case of home-schooling (*n* = 17), participants were assigned distance values of zero as their residence and school are one and the same. A pedestrian path could not be found for a small number of participants (*n* = 8). This was because the school and/or residence were located in a rural setting which could not be reached without the use of roads designated as motorways. These participants were excluded from analysis investigating associations with distance to school.

*School route topography* was calculated by intersecting the polyline routes generated when measuring distance to school with a digital elevation model (DEM) at one metre resolution. The Path Slope toolbox was used for the topography calculations. This toolbox works by segmenting the route by the DEM and calculating the slope for each individual segment. Once the slope value for each segment was calculated, they were assigned a value of either “0” or “1”, where segments with a slope <5% are assigned a value of “0” and those whose slope exceeds the 5% threshold are given a value of “1”. For each route, the total route length (TRL) is provided when the route was created within the GIS software, while the segments assigned a value of “1” are summed to provide a total steep route length (TSRL). The percentage of the route exceeding the defined threshold is calculated as TSRL/TRL.

##### Physical Activity

Physical activity was objectively assessed using GT3X+ ActiGraph accelerometers (Actigraph, Pensacola, FL, USA) [51,52,53]. Accelerometer count thresholds of Evenson et al. [54] were employed to classify time spent in MVPA. Non-wear time was classified as 60 min or more of consecutive zero counts [55]. At least seven hours of data were required for a valid day; participants with three or more valid days were included in analyses [56]. Percentage of time in MVPA was treated as a continuous variable in mediation analyses.

### 2.3. Data Analysis

Analyses were conducted for the total sample, and males and females separately. Characteristics of males and females were compared using t tests for continuous variables and chi-squared tests for categorical variables. Continuous variables are presented as means and standard deviation and categorical variables as counts and frequencies as percentage (Table 1).

We tested whether a binary outcome for IDF risk factors (none versus some) was associated with neighbourhood built environment measures using logistic regression with and without adjustment for household SES. We also tested if FFM as a continuous variable was associated with neighbourhood built environment measures with linear modelling with and without adjustment for SES. Mediation effects were examined with neighbourhood built environment factors (x, independent), FFM and metabolic factors (y, dependent), and measures of physical activity (m, potential mediators) using R statistical package “mediation” [57]. R statistical package [58] was used for all data analyses.

## 3. Results

Participant characteristics are provided in Table 1. There were significant differences in metabolic risk factors, physical activity, and time spent sedentary between males and females. Males had much higher risk for metabolic syndrome. Time spent in moderate to vigorous physical activity was minimal (<8%) for both males and females. Overall, 51% of participants had one or more IDF metabolic risk factors; one third of females and two thirds of males (*p* < 0.001). No significant differences in neighbourhood built environment variables or household-level SES were found between males and females.

Association test results are provided in Table 2 and Table 3. Significant associations were observed between pedestrian connectivity and metabolic risk in males only. In both unadjusted and adjusted modelling, residing in a neighbourhood with one percentage point higher pedestrian connectivity was associated with a 4.8% increase in the chance of having any IDF metabolic risk factors (adjusted odds ratio = 1.048 (95%CI 1.003, 1.095)). Conversely, there was a significant positive relationship between neighbourhood pedestrian connectivity and FFM in males only, where one percentage point higher of pedestrian connectivity was associated with an increase of 0.24 (95%CI 0.01, 1.47) kg of FFM, although this relationship was attenuated after controlling for SES. The correlation (Pearson r) between neighbourhood pedestrian connectivity for males was 0.228 (95%CI 0.037, 0.403) and for females 0.127 (95%CI −0.071, 0.316).

## 4. Discussion

The aim of this study was to investigate associations between objectively-assessed built environment attributes and metabolic risk factors in adolescents of Pacific Islands ethnicity residing in Auckland, New Zealand, and to consider the possible mediating effect of moderate-to-vigorous physical activity and sedentary time in these relationships. In doing so, this study contributes to the knowledge base by using sensitive, specific, and objective measures of dependent, independent, and mediating variables with a unique, high-risk population. Differential findings were observed by metabolic outcome and sex. Residing in a neighbourhood with greater pedestrian connectivity was associated with higher odds of having one or more metabolic risk factors in males only. Conversely, greater pedestrian connectivity was associated with higher fat-free mass, again in males only, and household level SES status impacted this relationship.

This study is the first to consider the relationship between pedestrian network connectivity and adolescent metabolic health. The findings for the positive relationship with FFM align with previous work with adults where residing in more “walkable” environments has been associated with improved metabolic health including lower blood pressure, lower BMI, higher HDL, and reduced risk of diagnosed diabetes mellitus [32,33,34]. There is a paucity of research examining such relationships in adolescents, and the evidence base is inconsistent. One New Zealand study including adolescents and adults aged 15–65 years found negative associations between street connectivity and body size (BMI and waist circumference) [35]. Findings were not stratified by age, so it was unclear whether findings differed from the overall results for the adolescent participants. A US study reported almost no associations between built environment features and body mass for a large sample of adolescents, with the exception of sidewalk completeness [36]. Contrary to expectations (and conceptually in disagreement with the current study findings related to FFM), sidewalk completeness was positively associated with BMI z-score. 

At first glance, the contrasting findings in the current study seem perplexing. However when considering precursors to IDF metabolic risk criteria and FFM, some plausible explanations arise. Importantly, nutrition behaviours and the nutrition built environment were not examined in this study. Nutrition plays an essential role in a range of metabolic risk factors. It is likely the neighbourhood nutrition environment may relate to both nutrition behaviours and metabolic health [32,59]. Increased access to suboptimal nutrition environments (e.g., through increased pedestrian connectivity) may encourage unhealthy nutrition behaviours, ultimately leading to increased metabolic risk [60]. On the other hand, greater FFM can exist in the presence of other metabolic risk factors. To some extent being heavier can positively impact the development of FFM through greater loading during physical activities. It could be hypothesised that greater pedestrian connectivity leads to improved FFM profiles through enabling walking as an activity in its own right, and as a mode of transport to destinations. While these destinations might still include suboptimal nutrition environments, they are also likely to include places to be physically active (as evidenced in the accessibility to open spaces in this study) and thus increase FFM. 

Numerous potential reasons exist for the differences observed in this small evidence base, the most obvious being use of different independent and dependent variables, and considerably different socio-demographic characteristics of study populations. Indeed, even within the study of Duncan et al. [36], differential findings were observed by ethnicity, whereby bus stop density was positively related to a higher BMI z-score in adolescents of European ethnicity, and retail destinations and BMI z-scores were inversely related for those of Asian ethnicity. 

Buffer choice for calculating built environment variables may also play a role in these divergent findings. Physical activity research has shown differential relationships are found between the built environment and adolescent MVPA when using different neighbourhood buffers (and for different variables). For example, physical activity facilities within 3 km buffers and intersection density within 1 km buffers exhibited the most consistent associations with activity in one study [61]. Another study examined the sensitivity of findings to the spatial scale of analysis (i.e., 400 m and 800 m street network buffers), and significant associations were found for the 800 m buffer [36]. The BEANZ study found associations for 2 km buffer only (and not for shorter buffer distances) [24]. A US study reported a 0.75 mile (1207 m) buffer as optimal to represent older female adolescents’ walkable neighbourhood [62]. The 1200 m buffer was deemed an appropriate compromise between the 800 m and 2 km buffer for the current study, and enabled comparability with some earlier research. 

Similar to Duncan et al. [36], no other significant relationships between neighbourhood built environment variables and metabolic health were observed in this study. Objective measurement of the built environment is generally seen as a strength due to the reduced risk of self-report bias, for example through issues with recall, comprehension, and social desirability [63,64]. A growing body of evidence is demonstrating the mismatch between objectively measured built environments and resident perceptions of those environments [65]. Evidence is also mounting for the differential relationships observed when objective or perceived neighbourhood measures are examined in relation to human behaviors and health outcomes [65]. Adolescent neighbourhood safety perceptions and parental directives may play an important role in opportunities for adolescents to accumulate physical activity for metabolic health [23,24]. At the individual and family level, neighbourhood perceptions may mediate the environment-activity-metabolic health relationship, particularly considering aesthetics and amenities of destinations [66], personal safety, and safety from traffic [67,68,69,70,71].

### Strengths and Limitations

This study improves on earlier research by considering pedestrian network connectivity within the neighbourhood buffer [30]. It is worth noting that this measure is not without criticism. Early work in this area demonstrated small but significant correlations between self-reported active transport and both pedestrian network intersection density (*R* = 0.123 for 500 m buffer; *R* = 0.142 for 1000 m buffer) and metric reach (*R* = 0.120 for 500 m buffer; R = 0.132 for 1000 m buffer; all *p* < 0.001) [72]. The authors concluded intersection density and metric reach may be the most appropriate pedestrian network measures to employ in future indices of walkability and suggested that the PedShed performed poorly (R = 0.066, *p* = 0.035 for 500 m buffer; R = 0.102, *p* = 0.001 for 1000 m buffer). While measurements generated around individual addresses are an improvement on area-level measures, it is also worth noting that this approach is limited in understanding exact exposures to environments. Approaches such as generating individualised activity spaces may be worthwhile in future research [73]. 

The definition of metabolic syndrome can also be criticised particularly in relationship to the criteria of waist and blood pressure. These Pacific youth are of exceptional size compared to other populations. Along with higher body weight and height for age it would be expected that blood pressure would also be higher as blood pressure is higher in taller people [74]. In addition, for the same body mass index as European, Pacific children [75] and adults [76] have less fat and more fat free mass. Given the study population were of Pacific Islands ethnicity, findings may not be generalisable to other population groups.

This research focused on the neighbourhood physical activity environment only, drawing from the body of literature demonstrating (a) significant relationships between residing in a more walkable environment and physical activity, and (b) physical activity behaviours and metabolic health. Other factors may also contribute to adolescent physical activity and metabolic health [18], including the home environment [77], individual socio-cognitive factors [78], and adolescents’ social contexts [79,80,81], and the social environment. Nutrition behaviours and the nutrition built environment also play an important role in metabolic health, however neither were considered in the current study. Future research examining a broader suite of environmental characteristics across the socio-ecological spectrum in relation to adolescent metabolic health, while challenging, would be worthwhile. Due to the cross-sectional approach employed in the current study, causality cannot be inferred. Future research would benefit from examining longitudinal exposures to neighborhood environments in order to understand the role of repeated exposure (or otherwise) to specific environmental features in developing health risk [82,83]. 

## 5. Conclusions

This study is the first to provide evidence for the importance of pedestrian network connectivity in relation to adolescent metabolic health. Residing in a neighbourhood with greater pedestrian network connectivity was associated with increased metabolic risk in males of Pacific Islands ethnicity. It is likely this scenario reflects greater exposure to suboptimal food environments. This suggests a need for planning and policy approaches to reduce easy access to and availability of nutrient poor, unhealthy foods and improved access to unprocessed healthy food options. Future research is required to expand the limited evidence base in neighbourhood environments and adolescent metabolic health.

## Figures and Tables

**Table 1:** Participant and neighbourhood characteristics.

Variable	All *n* = 204		Females *n* = 100		Males *n* = 104		*p* Value
	Mean *(or n)*	SD *or %*	Mean *(or n)*	SD *or %*	Mean *(or n)*	SD *or %*	
**Age (years)**	14.90	0.45	14.92	0.47	14.88	0.43	0.481
**Weight**	83.6	23.1	81.3	20.6	85.8	25.2	0.156
**Height**	171.0	7.8	167.0	5.6	175.0	7.1	<0.00001
**Fat free mass (kg)**	58.5	13.7	53.9	12.6	63.0	13.2	**<0.001**
Metabolic risk							
**IDF risk factors**							
***Waist circumference (cm)***	86.7	17.7	84.6	16.2	88.7	18.8	0.098
***Triglycerides (mmol/L)***	1.03	0.46	0.99	0.41	1.07	0.51	0.239
***HDL-C (mmol/L)***	1.33	0.27	1.37	0.27	1.29	0.26	**0.026**
***Systolic blood pressure (mmHg)***	121	10	118	9	124	11	**<0.001**
***Diastolic blood pressure (mmHg)***	71	7	72	6	71	7	0.313
***Glucose (mmol/L)***	5.15	0.84	5.05	0.82	5.23	0.85	0.117
***No IDF risk factors n (%)***	100	49.0%	64	64.0%	36	34.6%	**<0.00001**
***One or more IDF risk factors***	104	51.0%	36	36.0%	68	65.4%	
***One***	61	29.9%	23	23.0%	38	36.5%	
***Two***	35	17.2%	11	11.0%	24	23.1%	
***Three***	5	2.5%	1	1.0%	4	3.8%	
***Four***	3	1.5%	1	1.0%	2	1.9%	
							
Physical activity							
**% of time in MVPA**	5.56	3.53	3.66	2.22	7.32	3.61	**<0.001**
**% of time sedentary**	62.70	8.11	64.64	7.24	60.91	8.50	**0.002**
Neighbourhood built environment							
**Net Residential density × 10^2^**	10.74	2.08	10.74	2.05	10.73	2.12	0.953
**Land use mix × 10^2^**	21.51	14.74	23.16	15.38	19.93	13.99	0.118
**Street network connectivity**	8.76	4.94	9.02	4.69	8.51	5.18	0.462
**Pedestrian network connectivity, %**	38.80	11.56	38.47	12.13	39.12	11.02	0.689
**Accessibility to open spaces, %**	51.82	41.85	50.37	34.24	53.21	48.17	0.627
**Accessibility to destinations**	42.12	9.17	42.15	8.71	42.09	9.63	0.966
**Traffic speed exposure, %**	16.25	20.72	16.19	17.79	16.30	23.27	0.970
**Distance to school, km**	3.85	3.22	4.00	3.25	3.71	3.19	0.532
**School route topography**	5.67	9.41	5.20	7.76	6.13	10.76	0.479
Household socio-economic status (number of deprivations reported)							0.092
**1**	36	17.6%	21	21.0%	15	14.4%	
**2**	43	21.1%	21	21.0%	22	21.2%	
**3–4**	67	32.8%	27	27.0%	40	38.5%	
**5–8**	32	15.7%	21	21.0%	11	10.6%	
**Missing/incomplete/not reported**	26	12.7%	10	10.0%	16	15.4%	

Notes: % = percent; HDL-C = High-density lipoprotein cholesterol; IDF = International Diabetes Federation; MVPA = moderate-to-vigorous physical actrivity; *n* = number of participants; *p*-value = test for significant difference between males and females; SD = standard deviation. Bold indicates significant difference between males and females at *p* < 0.05.

**Table 2:** Logistic regression analyses examining associations between neighbourhood built environment factors and metabolic risk (any metabolic risk factor present).

Model	All *n* = 204		Females *n* = 100		Males *n* = 104	
Neighbourhood Exposure Variable	Odds Ratio (95% CI)	*p* Value	Odds Ratio (95% CI)	*p* Value	Odds Ratio (95% CI)	*p* Value
Unadjusted						
**Net Residential density × 10^2^**	0.954 (0.835, 1.089)	0.485	0.986 (0.808, 1.205)	0.893	0.918 (0.754, 1.117)	0.392
**Land use mix × 10^2^**	0.995 (0.977, 1.014)	0.606	0.983 (0.955, 1.011)	0.233	1.020 (0.988, 1.054)	0.228
**Street network connectivity**	0.981 (0.927, 1.037)	0.497	1.043 (0.956, 1.139)	0.346	0.942 (0.871, 1.019)	0.138
**Pedestrian network connectivity, %**	1.011 (0.987, 1.036)	0.36	0.981 (0.949, 1.045)	0.271	**1.047** **(1.008, 1.088)**	**0.018**
**Accessibility to open spaces, %**	1.000 (0.994, 1.007)	0.897	0.992 (0.977, 1.007)	0.296	1.004 (0.994, 1.014)	0.469
**Accessibility to destinations**	1.008 (0.978, 1.039)	0.604	0.975 (0.930, 1.022)	0.296	1.038 (0.993, 1.085)	0.096
**Traffic speed exposure, %**	1.004 (0.991,1.018)	0.567	0.998 (0.976, 1.022)	0.895	1.009 (0.988, 1.029)	0.409
**Distance to school, km**	1.003 (0.920, 1.093)	0.947	1.087 (0.959, 1.232)	0.190	0.941 (0.831, 1.065)	0.337
**School route topography**	1.003 (0.974, 1.033)	0.817	0.943 (0.879, 1.012)	0.106	1.029 (0.982, 1.078)	0.234
Adjusted for household socio-economic status						
**Net Residential density × 10^2^**	0.979 (0.848, 1.130)	0.774	0.967 (0.784, 1.193)	0.755	0.992 (0.802, 1.228)	0.944
**Land use mix × 10^2^**	0.993 (0.973, 1.014)	0.532	0.986 (0.957, 1.015)	0.347	1.010 (0.973, 1.048)	0.599
**Street network connectivity**	0.978 (0.920, 1.040)	0.48	1.026 (0.936, 1.126)	0.581	0.951 (0.871, 1.039)	0.267
**Pedestrian network connectivity, %**	1.018 (0.992, 1.045)	0.183	0.997 (0.961,1.034)	0.853	**1.048 (1.003, 1.095)**	**0.037**
**Accessibility to open spaces, %**	1.000 (0.944, 1.007)	0.892	0.992 (0.976, 1.008)	0.322	1.005 (0.994, 1.016)	0.347
**Accessibility to destinations**	1.016 (0.983, 1.050)	0.353	0.998 (0.945, 1.055)	0.950	1.023 (0.977, 1.072)	0.331
**Traffic speed exposure, %**	1.008 (0.993, 1.023)	0.318	1.000 (0.973, 1.026)	0.975	1.012 (0.989, 1.035)	0.314
**Distance to school, km**	0.987 (0.896, 1.088)	0.798	1.018 (0.880, 1.179)	0.807	0.962 (0.839, 1.104)	0.585
**School route topography**	1.000 (0.970, 1.033)	0.981	0.927 (0.854, 1.006)	0.070	1.039 (0.981, 1.101)	0.194

Note: bold signifies significant at *p* < 0.05.

**Table 3:** Linear regression analyses examining associations between neighbourhood built environment factors and fat free mass.

Model	All *n* = 204		Females *n* = 100		Males *n* = 104	
Neighbourhood Exposure Variable	β Coefficient (95% CI)	*p* Value	β Coefficient (95% CI)	*p* Value	β Coefficient (95% CI)	*p* Value
Unadjusted						
**Net Residential density × 10^2^**	−0.030 (−0.937, 0.878)	0.949	0.880 (−0.330, 2.090)	0.157	−0.829 (−2.030, −0.662)	0.178
**Land use mix × 10^2^**	−0.052 (−0.180, 0.076)	0.429	−0.052 (−0.214, 0.110)	0.532	0.021 (−0.162, 0.204)	0.823
**Street network connectivity**	0.004 (−0.378,0.387)	0.982	0.323 (−0.206, 0.853)	0.235	−0.167 (−0.661, 0.328)	0.511
**Pedestrian network connectivity, %**	0.155 (−0.007, 0.318)	0.062	0.055 (−0.152, 0.262)	0.604	**0.242 (0.014, 1.469)**	**0.040**
**Accessibility to open spaces, %**	−0.019 (−0.065, 0.026)	0.403	−0.058 (−0.130, 0.015)	0.121	−0.005 (−0.058, 0.049)	0.855
**Accessibility to destinations**	0.057 (−0.150, 0.263)	0.591	−0.063 (−0.350, 0.225)	0.671	0.154 (−0.111, 0.419)	0.257
**Traffic speed exposure, %**	0.040 (−0.051, 0.132)	0.391	−0.020 (−0.161, 0.1210	0.780	0.077 (−0.033, 0.187)	0.175
**Distance to school, km**	−0.430 (−1.020, 0.156)	0.152	−0.202 (−0.976, 0.573)	0.611	−0.517 (−1.320, 0.281)	0.207
**School route topography**	−0.036 (−0.239, 0.168)	0.733	−0.137 (−0.475,0.200)	0.426	−0.035 (−0.274, 0.203)	0.771
Adjusted for household socio-economic status						
**Net Residential density × 10^2^**	0.143 (−0.864, 1.150)	0.782	0.901 (−0.419, 2.220)	0.185	−0.622 (−2.010, 0.768)	0.383
**Land use mix × 10^2^**	−0.063 (−0.210, 0.085)	0.406	−0.058 (−0.238, 0.122)	0.528	−0.017 (−0.255, 0.220)	0.886
**Street network connectivity**	0.067 (−0.361, 0.495)	0.759	0.446 (−0.135, 1.030)	0.136	−0.139 (−0.717, 0.440)	0.640
**Pedestrian network connectivity, %**	0.120 (−0.063, 0.303)	0.201	0.029 (−0.202, 0.260)	0.807	0.232 (−0.037, 0.501)	0.094
**Accessibility to open spaces, %**	−0.017 (−0.065, 0.031)	0.487	−0.056 (−0.134, 0.023)	0.166	0.012 (−0.048, 0.071)	0.707
**Accessibility to destinations**	0.020 (−0.213, 0.254)	0.865	−0.095 (−0.439, 0.249)	0.589	0.086 (−0.214, 0.386)	0.575
**Traffic speed exposure, %**	0.069 (−0.032, 0.170)	0.184	−0.003 (−0.170, 0.164)	0.974	0.086 (−0.035, 0.206)	0.167
**Distance to school, km**	−0.304 (−0.989, 0.381)	0.385	−0.115 (−1.060, 0.0827)	0.811	−0.396 (−1.320, 0.527)	0.403
**School route topography**	−0.052 (−0.272, 0.167)	0.640	−0.174 (−0.535, 0.187)	0.347	−0.018 (−0.282, 0.246)	0.892

Note: bold signifies significant at *p* < 0.05.
